# Vitamin C Deficiency May Delay Diet-Induced NASH Regression in the Guinea Pig

**DOI:** 10.3390/antiox11010069

**Published:** 2021-12-28

**Authors:** Josephine Skat-Rørdam, Kamilla Pedersen, Gry Freja Skovsted, Ida Gregersen, Sara Vangsgaard, David H. Ipsen, Markus Latta, Jens Lykkesfeldt, Pernille Tveden-Nyborg

**Affiliations:** 1 Department of Veterinary and Animal Sciences, Section of Experimental Animal Models, University of Copenhagen, Grønnegårdsvej 15, DK-1870 Frederiksberg, Denmark; jsr@sund.ku.dk (J.S.-R.); kamilla.pedersen@sund.ku.dk (K.P.); gryfreja@sund.ku.dk (G.F.S.); idagregersen@sund.ku.dk (I.G.); saravangsgaard@sund.ku.dk (S.V.); jopl@sund.ku.dk (J.L.); 2 Global Obesity and Liver Disease Research In Vivo Pharmacology DK II, Novo Nordisk A/S, Novo Nordisk Park 1, 2760 Måløv, Denmark; DVIE@novonordisk.com; 3 Global Obesity & Liver Disease Research, Global Drug Discovery, Novo Nordisk A/S, Novo Park 1, 2670 Måløv, Denmark; mrlq@novonordisk.com

**Keywords:** non-alcoholic fatty liver disease (NAFLD)/steaotohepatitis (NASH), vitamin C, guinea pig model

## Abstract

Oxidative stress is directly linked to non-alcoholic fatty liver disease (NAFLD) and the progression to steaotohepatitis (NASH). Thus, a beneficial role of antioxidants in delaying disease progression and/or accelerating recovery may be expected, as corroborated by recommendations of, e.g., vitamin E supplementation to patients. This study investigated the effect of vitamin C deficiency—often resulting from poor diets low in fruits and vegetables and high in fat—combined with/without a change to a low fat diet on NAFLD/NASH phenotype and hepatic transcriptome in the guinea pig NASH model. Vitamin C deficiency per se did not accelerate disease induction. However, the results showed an effect of the diet change on the resolution of hepatic histopathological hallmarks (steatosis, inflammation, and ballooning) (*p* < 0.05 or less) and indicated a positive effect of a high vitamin C intake when combined with a low fat diet. Our data show that a diet change is important in NASH regression and suggest that a poor vitamin C status delays the reversion towards a healthy hepatic transcriptome and phenotype. In conclusion, the findings support a beneficial role of adequate vitamin C intake in the regression of NASH and may indicate that vitamin C supplementation in addition to lifestyle modifications could accelerate recovery in NASH patients with poor vitamin C status.

## 1. Introduction

Redox imbalance and consequent oxidative stress is an important driver of hepatocellular damage in non-alcoholic fatty liver disease (NAFLD) and the subsequent progression to steaotohepatitis (NASH) and fibrosis; a primary indicator for liver transplantation [1,2]. A causal connection to a chronically high calorie intake (in the form of fat, cholesterol, and sugars) combined with a sedentary lifestyle ties NAFLD to the broadly termed “lifestyle associated diseases”, hereby representing the hepatic consequence of metabolic dysfunction [3,4]. In this way, closely linked to dyslipidemia and metabolic stress, an imposing state of redox imbalance and resulting oxidative stress in NAFLD induces hepatocellular damage and release of inflammatory cytokines and profibrotic factors, driving disease progression with serious consequences for liver function and patient health [2,5].

By quenching free radicals, antioxidants are pivotal in maintaining intracellular redox balance and are likely to play an important role in NAFLD/NASH progression and resolution. In agreement, vitamin E (vitE) supplementation has been shown to improve NASH histology in experimental animal models [6,7,8] and in humans [9], including large clinical trials (PIVENS (placebo vs. 800IU vitE or 30 mg Pioglitazone) [10] and TONIC (placebo vs. 800IU vitE or 1000 mg Metformin) [11]). Though mechanisms are not completely disclosed, vitE has been included as part of the treatment guidelines for some NAFLD patients [12,13,14,15,16].

However, other antioxidants may also play a role in NAFLD progression and resolution. Of these, vitamin C (vitC) may be the most important. VitC is considered the most important antioxidant in the blood and the only antioxidant capable of preventing lipid oxidation [17]. Moreover, decreased vitC levels are reported in NAFLD patients and are associated with several NAFLD/NASH co-morbidities such as obesity, cardiovascular diseases, and type 2 diabetes, though a causal relationship between vitC deficiency and accelerated NAFLD/NASH progression has not yet been established [18,19,20,21,22,23,24]. Acting as an electron donor and situated low in the free radical ‘pecking order’, ascorbate (the reduced form of vitC) provides reducing equivalents to diminish oxidative stress, but also to recycle other antioxidants such as vitE [25,26]. In addition, vitC acts as a cofactor in multiple enzymatic reactions, including in the formation of mature collagen and in the regulation of gene expression through DNA methylation, supporting a role in NAFLD progression [27,28,29].

Like humans, guinea pigs depend on a dietary vitC intake, contrary to most other mammals that have an endogenous vitC synthesis [30]. Moreover, guinea pigs develop NASH with hepatic fibrosis when subjected to a high fat diet, and share a high degree of similarity with the human NAFLD/NASH transcriptome and accompanying NASH histopathology [31,32,33,34,35]. Together, this supports a uniquely high translational potential of the guinea pig NASH model including a high predictability of mechanistic responses related to disease phenotype as well as vitC homeostasis.

This study investigated the potential burden of vitamin C deficiency—often resulting from a poor diet low in fruits and vegetables and high in fat—on the guinea pig NAFLD/NASH phenotype and hepatic transcriptome. The consequences of high versus low vitC intake on diet-induced NASH progression were assessed, as well as effects of vitC supplementation on subsequent NASH resolution combined with or without a low fat/low cholesterol diet, as recommended by clinical guidelines to patients.

## 2. Materials and Methods

Animal experiments were approved by the Danish Experimental Animal Inspectorate (License No: 2018-15-0201-01591) and in accordance with European legislation on animal experimentation (Directive 2010/63/EU on the protection of animals used for scientific purposes).

Eighty female Hartley guinea pigs between 301 and 350 g (Charles River Laboratory, Lyon, France) were equipped with an 1.4 mm subcutaneous microchip (E-vet, Haderslev, Denmark) and allocated by randomization into three weight stratified groups upon arrival, all receiving a low fat high vitC diet (LFH) (control) diet. Following one week of acclimatization, high fat diets were introduced gradually over a 5-day period, establishing the three experimental groups for the 16 weeks of NASH induction. Groups consisted of the following: low fat-high vitC (control) (LFH, *n* = 16) (3.8% fat, 0% cholesterol, 0% sucrose, 2000 mg vitC/kg feed); high fat-high vitC (HFH, *n* = 16) (20% fat, 15% sucrose, 0.35% cholesterol, 2000 mg vitC/kg feed); and high fat-low vitC (HFL, *n* = 48) (20% fat, 15% sucrose, 0.35% cholesterol, 50 mg vitC/kg feed). For a more detailed diet composition, see Table S1.

HFH and HFL groups received feed ad libitum, whereas the LFH group was pair fed to the HFH group, ensuring the LFH group as a lean and metabolic healthy control with comparable vitC intake [36]. During the 16-week induction period, two animals (one from the LFH group and one from the HFL group) were euthanized because of a lack of sufficient weight gain, consequently reaching the humane endpoint of a maximum of 20% lower body weight compared with the group average. Neither of the animals displayed any signs of underlying disease in vivo or in the post mortem necropsy.

**Figure 1:**
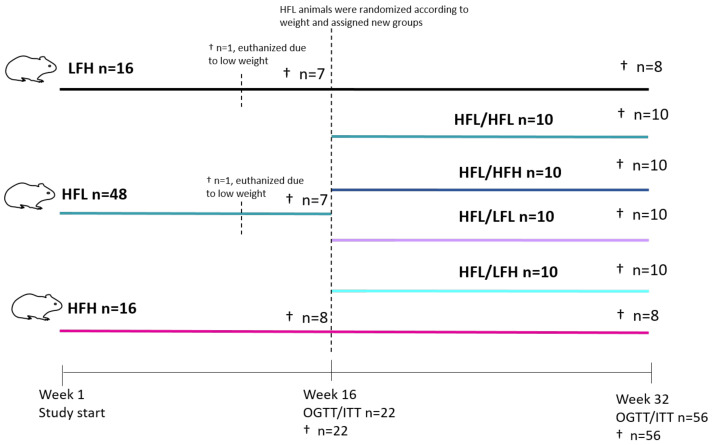
Study overview. Eighty guinea pigs were weight stratified into HFH, HFL, or LFH groups. One animal from each of the HFL and LFH groups was euthanized owing to weight loss. Following 16 weeks on diets, a subset were selected for OGTT/ITT and subsequent euthanization (HFL and LFH *n* = 7; HFH *n* = 8). The remaining animals in LFH and HFH groups continued on diets, whereas those in the HFL group (*n* = 40) were weight stratified into HFL/HFL, HFL/HFH, HFL/LFL, or HFL/LFH. Animals continued on diets until week 32, where all were subjected to OGTT and ITT before termination. HFL: high fat low vitC; HFH: high fat high vitC; LFH: low fat high vitC; LFL: low fat low vitC; ITT: insulin tolerance test; OGTT: oral glucose tolerance test; vitC: vitamin C.

After 16 weeks on diets, oral glucose tolerance test (OGTT) and insulin tolerance test (ITT) were performed on a subset of animals from each group (selected by randomization and stratified by bodyweight) (LFH *n* = 7; HFL *n* = 7 and HFH *n* = 8) prior to euthanization (see Figure 1 for study overview). The remaining animals in LFH and HFH groups (*n* = 8/group) continued on their diet until study termination. Animals in the HFL group (*n* = 40) were randomized into four weight-stratified intervention groups (*n* = 10/group) to receive one of the following diets in a 2 by 2 factorial design: high fat-low vitC (HFL/HFL); high fat-high vitC (HFL/HFH); low fat-high vitC (HFL/LFH); or a low fat-low vitC (HFL/LFL) (LFL: 3.8% fat, 0% cholesterol, 0% sucrose, 50 mg vitC/kg feed). The 50 mg vitC/kg feed was obtained by titrating feed with 100 mg vitC/kg with feed containing 0 mg vitC/kg feed. The new diets were introduced gradually over 5 days. The HFL/LFH group was pair-fed to the HFL/HFH group, and the HFL/LFL group was pair fed to the HFL/HFL group to ensure equal intake of vitC. After 16 weeks on diets, OGTT and ITT were performed on all animals (*n* = 56), after which all animals were euthanized.

All diets were chow based with vitC provided in the form of phosphorylated ascorbate (Stay-C) and content confirmed by postproduction analysis (Ssniff Spezialdiäten, Soest, Germany). Feed intake was measured biweekly in each group by subtracting the amount of feed remaining with the amount given the previous day. All groups had ad libitum access to water and access to fixed amounts of hay throughout the study period. Body weights were measured once weekly and animal welfare was monitored daily by caretakers. No changes in behavior or clinical indications of disease or severe vitC deficiency (scurvy) were recorded.

### 2.1. Oral Glucose Tolerance Testing (OGTT)

All animals were semi-fasted (allowed access to hay and water) 12 h prior to testing. Testing at week 16 (*n* = 22) was performed over the course of two days. Testing at week 32 was on *n* = 56 animals and performed over the course of 3 days. Animals were weight stratified and randomized within groups ensuring equal distribution across testing days. On the day of testing, animals were weighed and 2 g/kg of a 150% glucose solution was administered orally through a syringe and voluntary swallowing (not gavage). Micro (50 µmL) blood samples for glucose monitoring were obtained by puncturing the ear vein with a 27 G needle at time-points 0 (baseline), 30, 60, 90, and 120 min after glucose administration, as previously described [36,37]. Blood glucose was measured in duplicates by an Aviva Accu-chek glucometer (Roche A/S Diagnostics, Hvidovre, Denmark).

### 2.2. Insulin Tolerance Testing (ITT)

Animals were fasted and randomized as described for OGTT. On the day of testing, animals were injected subcutaneously with 0.5 U/kg insulin (Actrapid^®^ Novo Nordisk A/S, Bagsværd, Denmark) with a 27 G needle. Blood samples were obtained as described for OGTT at 0 (baseline), 30, 60, 90, and 120 min after insulin administration. At week 32, one animal in the HFL/LFH group was excluded owing to inaccurate dosing.

### 2.3. Euthanasia and Sampling

As euthanasia proceeded across five days, animals were block randomized according to the day and time of euthanasia within groups. All animals were semi-fasted overnight and pre-anesthetized with 1.25 mL/kg Zoletil-mix (125 mg Tiletamin, 125 mg Zolazapam (Zoletil 50 Virbac Laboratories, Carros, France) + 200 mg xylazin (Narcoxyl vet 20 mg/mL; Intervet International, Boxmeer, Holland) + 7.5 mg butorphanol (Torbugesic vet 10 mg/mL; Scanvet, Fredensborg, Denmark)). The anesthetized animal was then placed on isofluorane (3–5%) and, upon disappearance of inter-digital reflexes, intracardial blood was collected for vitC, free fatty acids (FFAs), triglycerides (TGs), total cholesterol (TG), alkaline phosphatase (ALP), alanine aminotransferase (ALT), and aspartate aminotransferase (AST), as previously described [35,36,38].

HbA1c was collected from intra-cardial blood in 10 µL Na-Heparinized End-to-End Vitrex^®^ Pipettes (Vitrex medical A/S, Herlev Denmark) and mixed with 1 mL Hemolyzing Reagent nr. 11488457 122 (Cobas, Roche diagnostics, Rotkreuz, Switzerland). The mixture was left to incubate on ice for 10 min, after which a maximum of 200 uL was transferred to a cobas cup (Sample cup micro 13/16, Roche Diagnostics, Mannheim, Germany) and stored at −20 °C until analysis on a Cobas 6000 (Roche Diagnostics, Berne, Switzerland), according to the manufacturer’s instructions.

Liver samples were collected as previously described [35,36]. The three liver sections collected for TG, TC, and vitC were immediately frozen on dry ice, and stored at −80 °C. The liver section collected for RNA-sequencing was immediately frozen in liquid nitrogen and stored at −80 °C.

### 2.4. Histology

Liver sections for histology were fixed in 10% formalin and paraffin embedded prior to slicing in 2–4 µm thick sections and staining with hematoxylin and eosin (H&E) or picrosirius red (PSR) with Weigert’s hematoxylin solution. All histopathological scorings were performed in a randomized and blinded manner, as previously described for the guinea pig model and according to Kleiner et al. [33,35,39,40]. The reliability of scoring was assessed by Cohen’s Kappa index [41,42]. The index was calculated by scoring 10 randomly selected sections in a blinded manner, then re-blinding and re-scoring and comparing the results to confirm scoring integrity (Kappa index). The observer was only allowed to continue with the scoring of all sections if Cohen’s Kappa values were ≥0.8 for the following categories: steatosis, ballooning, and fibrosis and >0.7 for inflammation. Steatosis, inflammation, and ballooning were evaluated on H&E-stained sections. Steatosis and ballooning were evaluated across the entire liver section, and scored as 0 (<5%), 1 (5%–33%), 2 (>33%–66%), 3 (>66%), and 0 (none), 1 (few), and 2 (many) respectively. For lobular inflammation, a lobule was defined as two portal areas and one central vein, and an inflammatory focus was defined as three or more inflammatory cells in close proximity. Inflammatory foci were counted in five separate lobules dispersed across the liver section and scored as 0 (no foci), 1 (<2), 2 (2–4), and 3 (>4). Fibrosis scoring and quantification was performed across the entire section on PSR-stained sections. Fibrosis was scored as 0 (none), 1 (perisinusoidal or periportal), 2 (perisinusoidal and periportal), 3 (bridging), and 4 (cirrhosis). For fibrosis quantification, the relative fibrosis area was obtained by quantifying the amount of collagen stained tissue in relation to the total amount of liver tissue, using the Visiopharm software (version 2020.08.4.9377, VisioPharm, Hørsholm, Denmark), and in accordance with quantification of fibrosis from PSR-stained sections in preclinical and human studies [43,44]. NAFLD activity score (NAS) was derived from the cumulative sum of steatosis, inflammation, and ballooning ranging from 0 to 8 [40].

### 2.5. Transcriptome Analysis

RNA was extracted from 10 mg liver tissue from randomly selected animals from each experimental group at the 32-week time point (LFH: *n* = 8, HFH: *n* = 8, HFL/HFL: *n* = 10, HFL/HFH: *n* = 6, HFL/LFH: *n* = 6, HFL/LFL: *n* = 6), using the RNeasy Lipid Tissue Mini Kit in accordance with the manufacturer’s instructions (Qiagen, Valencia, CA, USA). Twenty-five microliters of the purified RNA (200–500 ng/mL measured by nanodrop 2000 (Thermo Fisher, Waltham, MA, USA) was shipped to Novogene (Novogene, Cambridge, UK) for paired-end 175-nucleotide read length, 30 million reads per sample, sequencing on an Illumina NovaSeq 6000 platform (Illumina Inc., San Diego, CA, USA). The resulting FASTQ files were trimmed using the Trimmomatic software (version 0.38.0) [45]. Any reads shorter than 50 nucleotides were removed as well as any Illumina specific adaptors. Furthermore, the initial nine bases were removed from each read to avoid biased sequence composition. The trimmed reads were mapped to the Ensembl Cavia Porcellus genome Cav.Por.3.0 (Ensembl release 98, September 2019) with Hisat2 (version 2.1.0) [46]. Transcript assembly and quantification was performed using Stringtie and Stringtie merge (version 2.1.1) [47] with the Cav.Por.3 annotation as a guide. To improve annotation, BioMart (version 2.42.0) [48] was used to obtain human orthologues, as previously described [34]. Genes without annotation and total read counts <200 for all samples were excluded from further analysis. The final list of genes was then used as input for differential expression analysis with DESeq2 (version 1.26.0) [49]. Gene set enrichment analysis (GSEA) was performed on log2 fold change pre-ranked values, using the fgsea package in R [50]. Hallmark pathways used as input were obtained from the Molecular Signature Database (MSigDB) [51,52,53]. Human top 150 NASH-associated advanced fibrosis genes were identified from gene expression data (GSE49541) consisting of 40 patients with mild (F0-F1) and 32 patients with advanced NAFLD/NASH and F3–F4 fibrosis [54].

### 2.6. Statistics

All statistical analysis was performed in the GraphPad Prism version 9.0.1 (GraphPad Prism software, La Jolla, CA, USA), or R version 4.1.1 (R Core Team, 2021.08.10). All normally distributed data with equal variances among groups were analyzed by one-, two-, or three-way ANOVA, with repeated measures if applicable, and Dunnett’s or Tukey’s correction for multiple comparisons presented as means with standard deviation (SD). Data deviating from normality were log transformed, re-analyzed, and subsequently presented as medians with 25th and 75th quartiles. In cases of continued deviation, or for categorical data, analysis was performed using a non-parametric Kruskal–Walis test with a Dunn’s test for multiple corrections, and presented as medians with 25th and 75th quartiles. For differential expression analysis, differentially expressed genes (DEGs) were defined as genes with a Benjamini–Hockberg corrected *p*-value (q-value) <0.05. For GSEA, a q-value <0.1 was considered significant. Principle component analysis was performed on normalized and transformed values for each gene, unless otherwise stated.

## 3. Results

### 3.1. Body Weights and Energy Intake

**Figure 2:**
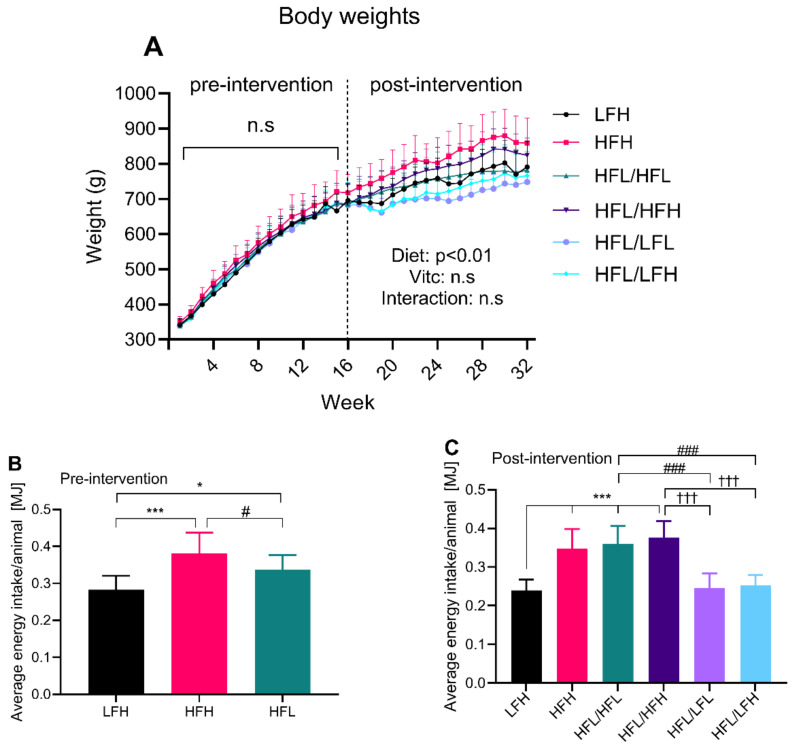
Body weights and energy intake. (**A**) Body weights presented as means ±SD; error bars are shown as one-sided on the graph. There were no differences in body weight in the pre-intervention period. For the post-intervention period, weights were analyzed by repeated measures three-way ANOVA, overall effects of factors (diet, VitC, and diet/vitC) depicted on the graph (*n* = 10). (**B**) Average energy intake/animal/day is presented as means ± SD, and was analyzed by one-way ANOVA with a Tukey’s multiple comparisons test, *n* = 13 weeks. (**C**) Average energy intake per animal is presented as means ± SD. LFH (control) was compared to all other groups by one-way ANOVA with a Tukey’s multiple comparisons test. In a separate analysis, all intervention groups were compared by one-way ANOVA with a Tukey’s multiple comparisons test, *n* = 13 weeks. Compared to LFH: * *p* < 0.05, *** *p* < 0.001; compared to HFL/HFL: # *p* < 0.05, ### *p* < 0.001; and compared to HFL/HFH: ††† *p* < 0.01. ns: not significant. HFH: high fat high vitC, HFL: high fat low VitC, LFH: low fat high vitC, LFL: low fat low vitC, vitC: vitamin C.

Body weights and energy intake were monitored pre- and post-intervention for all groups. While there were no differences in body weights in the pre-intervention period, Figure 2A shows a clear effect of diet (*p* < 0.01) in the post-intervention period and no effect of vitC. The HFH group had an increased energy intake compared with HFL in the pre-intervention period (Figure 2B), and displayed the highest body weight of all groups in the post-intervention period (week 16 and on). Interestingly, all intervention groups switching to a high vitC diet (HFL/HFH and HFL/LFH) show a weight pattern similar to the reference groups (HFH and LFH), rather than their low vitC counterparts, and despite similar energy intake between HFL/HFH and HFL/HFL, and between HFL/LFL and HFL/LFH (Figure 2C). Average energy intake per group was calculated as the mean of the bi-weekly measurements of feed intake once animals were completely adapted to their respective diets, hereby yielding *n* = 13 weeks pre- and post-intervention.

### 3.2. Glucose Homeostasis

**Figure 3:**
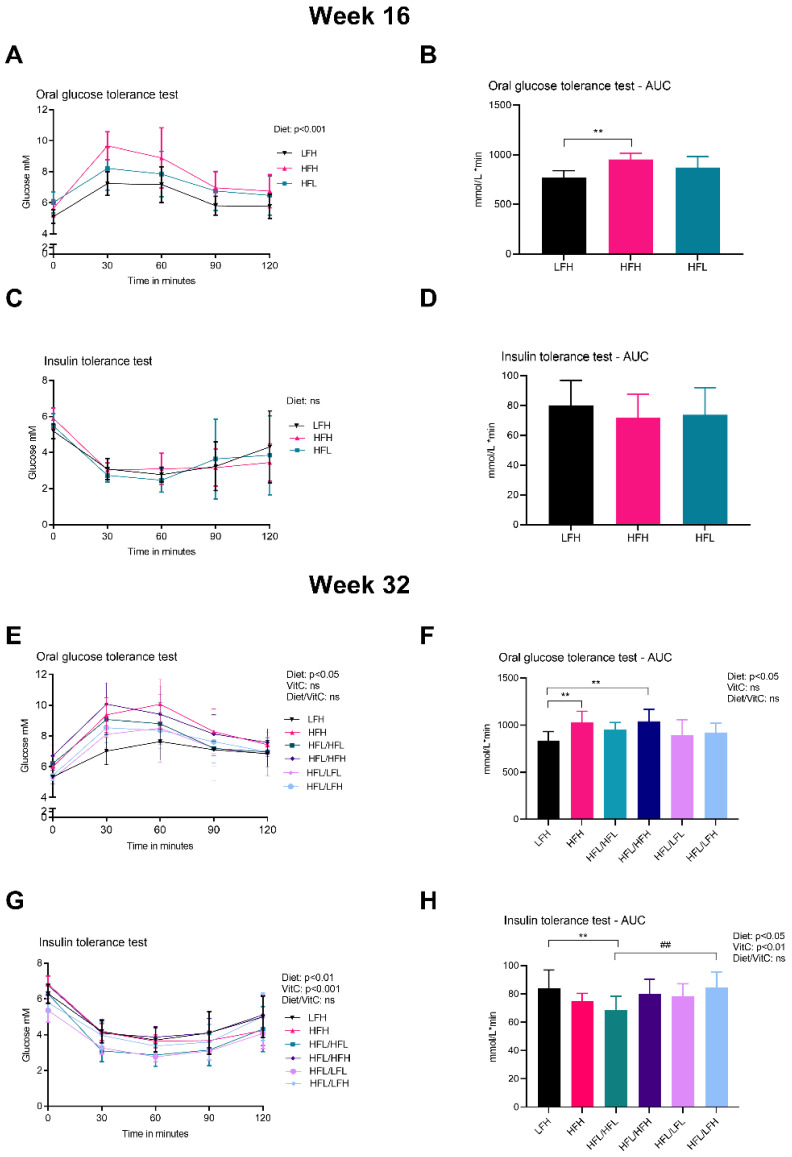
Glucose homeostasis. (**A**) OGTT at week 16. Log transformed data were analyzed by a two-way ANOVA (only results from the overall ANOVAs are shown). (**B**) AUC for OGTT at week 16. Data were analyzed by a one-way ANOVA with a Tukey’s test for multiple comparisons. (**C**) ITT at week 16. Log transformed data were analyzed by a two-way ANOVA (the overall ANOVA was not significant). (**D**) AUC for ITT at week 16. Normalized data were analyzed by a one-way ANOVA. (**E**) OGTT at week 32. Data were analyzed by a three-way ANOVA (only results from the overall ANOVA are shown). (**F**) AUC for OGTT at week 32. All groups were compared to LFH by a one-way ANOVA with a Dunnett’s test for multiple comparisons. In a separate analysis, intervention groups were analyzed by a two-way ANOVA with a Tukey’s multiple comparisons test. (**G**) ITT at week 32. Data were analyzed by a three-way ANOVA (only results from the overall ANOVA are shown). (**H**) AUC for ITT at week 32. Normalized data for all groups were compared to LFH by a one-way ANOVA with a Dunnett’s test for multiple comparisons. In a separate analysis, intervention groups were analyzed by a two-way ANOVA with a Tukey’s multiple comparisons test. All data are presented as means ± SD *n* = 7–10. ** *p* < 0.01 compared to LFH; ## *p* < 0.01 compared to HFL/HFL. ns: not significant. AUC: area under the curve, HFH: high fat high vitC, HFL: high fat low vitC, ITT: insulin tolerance test, LFL: low fat low vitC, LFH: low fat high vitC, OGTT: oral Glucose tolerance test, vitC: vitamin C.

The effect of diet and vitC on glucose homeostasis was assessed by OGTT, ITT, and determination of HbA1c concentration in plasma. At week 16 (pre-intervention), the OGTT response showed an overall effect of diet and increased AUC in HFH compared with LFH (*p* < 0.01) (Figure 3A,B). There was no difference in ITT responses or HbA1c (data not shown). At week 32 (post- intervention), HFH and HFL/HFH displayed the highest glucose peak levels and AUC of all groups (Figure 3E,F). The altered glucose homeostasis of these two high fat fed groups was supported by increased HbA1c levels compared with LFH (*p* < 0.01) and HFL/LFH (*p* < 0.001) (Table 1). Glucose measurements in the ITT showed a higher AUC in LFH and HFL/LFH compared with HFL/HFL animals (Figure 3H). ANOVA showed a significant effect of diet and of vitC on ITT glucose measurements (*p* < 0.001 and *p* < 0.01, respectively) with no diet/vitC interaction.

### 3.3. Plasma Markers

The lipid profile was assessed by measuring TG, TC, and FFA levels in plasma (pre-intervention values are presented in Supplementary Table S2). In line with the dietary cholesterol content (0.35%) in the high fat diets (HF groups), TC levels were clearly affected by diet (*p* < 0.001), and increased in all HF groups compared with animals on the low fat (no cholesterol) diets (LF groups) (*p* < 0.001) at week 32 (Table 2). TG and TC levels indicated an overall effect of vitC with increased levels in low vitC groups compared with their high vitC counterparts (*p* < 0.01) (Table 2). As expected, liver damage marker AST was increased in all HF groups compared with LF groups (*p* < 0.001), and ALT increased in HF compared with LF intervention groups (*p* < 0.01). Both ALT and AST levels were decreased in HFL/HFL compared with HFH (*p* < 0.05) (Table 2). Finally, vitC levels were significantly decreased in all low vitC groups (*p* < 0.001) (Table 2).

### 3.4. Liver Status

**Figure 4:**
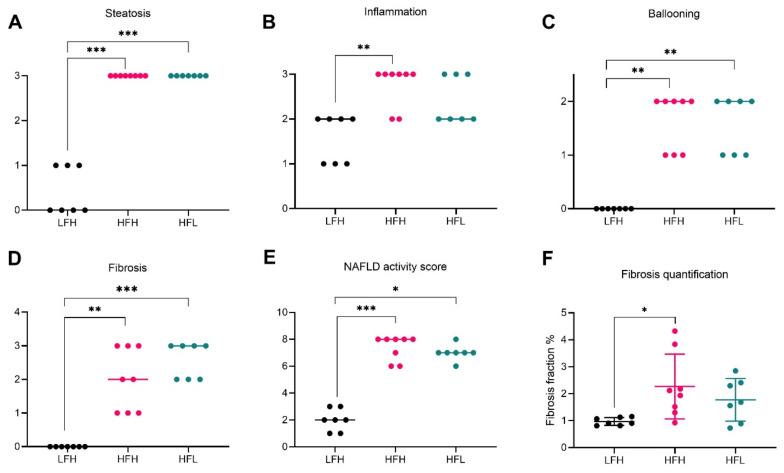
Hepatic histopathological scoring and fibrosis quantification at week 16. Data are represented as individual values (scores) with medians (**A**–**E**) or means ± SD (F) (*n* = 7–8/group). Semi-quantitative scoring results are depicted in (**A**) steatosis, (**B**) inflammation, (**C**) ballooning, and (**D**) fibrosis. (**E**) Cumulative NAFLD activity score for each animal. (**F**) Fibrosis fraction quantified by image analyses of picrosirius red stained sections and displayed as % of total tissue (section) area. Scoring was analyzed by a non-parametric Kruskal–Wallis with a Dunn’s multiple comparisons test, comparing all groups to LFH. For fibrosis quantification, LFH vs. all groups was analyzed by one-way ANOVA with Dunnett’s multiple comparisons test on log transformed data. Difference from LFH: * *p* < 0.05, ** *p* < 0.01, *** *p* < 0.001. HFH: high fat high vitC, HFL: high fat low vitC, LFH: low fat high vitC, VitC: vitamin C.

At the 16 weeks pre-intervention time point, NASH was successfully induced in both high fat diet groups (HFH and HFL), measured by increased steatosis, inflammation, ballooning, and consequently cumulated NAFLD activity scores compared with LFH animals (*p* < 0.001 for HFH and *p* < 0.05 for HFL) (Figure 4A–E). Fibrosis was also increased in both high fat groups (*p* < 0.01 and *p* < 0.001 in HFH and HFL respectively), with a median of F2 (range: F1–F4) stage in HFH and F3 stage (range F2–F3) in HFL animals. Significant differences in the histological distribution of fibrosis were only partially reflected by fibrosis quantification (*p* < 0.05 for HFH animals) (Figure 4F). Biochemical markers in the liver are presented in Supplementary Table S3.

Post-intervention hepatic TG and TC levels were increased in all HF groups compared with LF, hereby showing a clear effect of diet (*p* < 0.01), while there was no effect of vitC (Table 3). Post-intervention (32-week time-point) hepatic vitC levels were lower in the low vitC groups compared with high vitC groups, reflective of plasma values (*p* < 0.001) (Table 3). In line with previous findings from our group, HF groups demonstrated lower vitC levels compared with LF (*p* < 0.05) [35,36].

**Figure 5:**
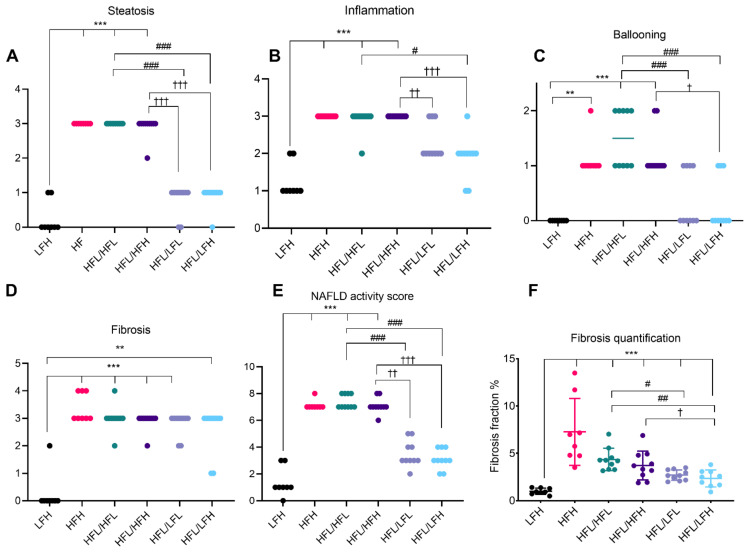
Hepatic histopathological scoring and fibrosis quantification at week 32. Data are represented as individual values (scores) with medians (**A**–**E**) or means ± SD (F) (*n* = 8–10/group). Semi-quantitative scoring results are depicted in (**A**) steatosis, (**B**) inflammation, (**C**) ballooning, and (**D**) fibrosis. (**E**). Cumulative NAFLD activity score for each animal. (**F**). Fibrosis fraction quantified by image analyses of picrosirius red stained sections and displayed as % of total tissue (section) area. Scoring was analyzed by a non-parametric Kruskal–Wallis with a Dunn’s multiple comparisons test, comparing all groups to LFH or to post-intervention groups in a separate analysis. For fibrosis quantification, LFH vs. all groups was analyzed by one-way ANOVA with Dunnett’s multiple comparisons test on log transformed data and, for intervention groups, data were analyzed by a two-way ANOVA with a Tukey’s test for multiple comparisons. Difference from LFH. ** *p* < 0.01, *** *p* < 0.001. Difference from HFL/HFL: # *p* < 0.05; ## *p* < 0.01; ### *p* < 0.001. Difference from HFL/HFH: † *p* < 0.05; †† *p* < 0.01; ††† *p* < 0.001. HFH: high fat high vitC, HFL: high fat low vitC, LFL: low fat low vitC, LFH: low fat high vitC, VitC: vitamin C.

**Figure 6:**
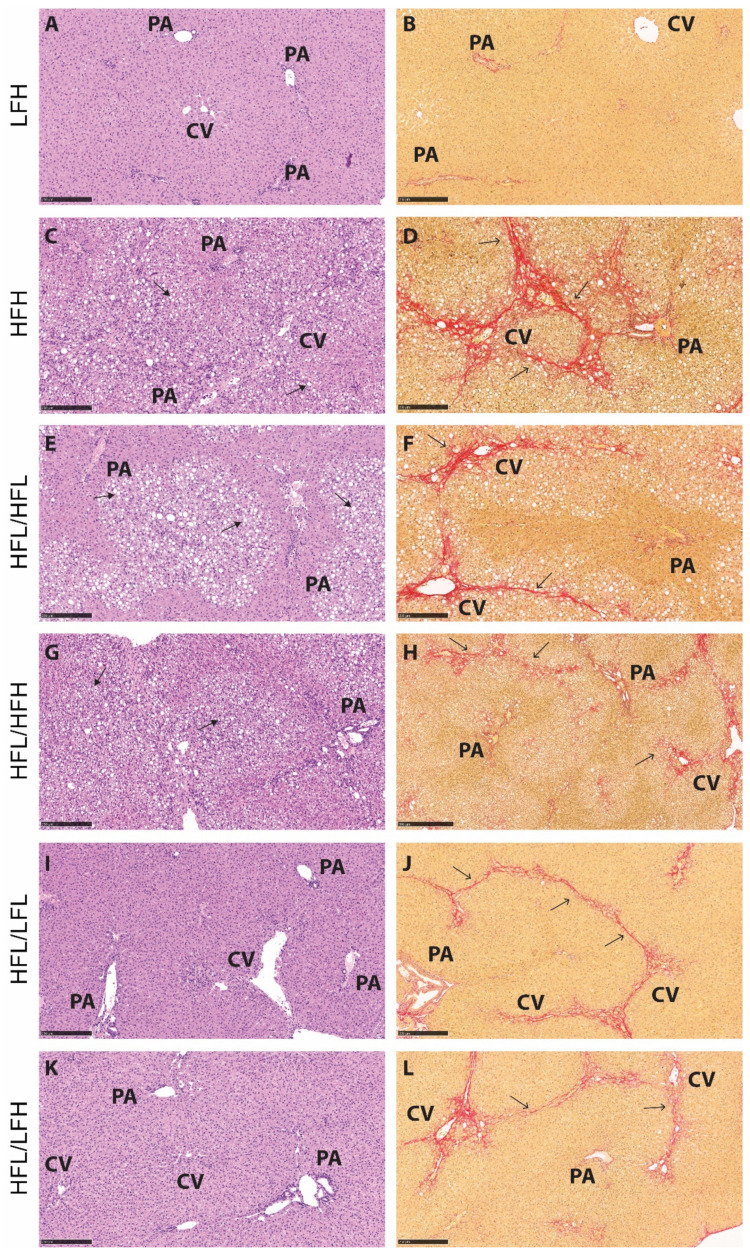
Representative histological images for each group at the 32-week time point. (**A**,**C**,**E**,**G**,**I**,**K**): Hematoxylin and eosin stain. Scale bar shows 250 µm. (**B**,**D**,**F**,**H**,**J**,**L**): Picrosirius red stain. Scale bar shows 250 µm. Solid arrows indicate lipid vacuoles (macro- and microvesicular steatosis), and open arrows indicate fibrosis (in red). CV: central vein, PA: portal area, HFL: high fat low vitC, HFH: high fat high vitC, LFL: low fat low vitC, LFH: low fat high vitC.

The effect of a high fat diet corresponds with hepatic histopathology of severe steatosis in HF groups compared with LF (*p* < 0.001) (Figure 5A and Figure 6C,E,G). The two HF intervention groups (HFL/HFL and HFL/HFH) did not exhibit significant differences in hepatic histopathology, and the median values were comparable to the HFH reference group (Figure 5 and Figure 6E–H). Both LF intervention groups showed decreased NAFLD activity score compared with HF intervention groups (HFL/HFH and HFL/HFL, *p* < 0.01 or less). Fibrosis quantification demonstrated decreased fibrosis in both LF intervention groups (HFL/LFH and HFL/LFL) compared with the HF groups, though this was not reflected in the scoring of the overall morphological distributional pattern of fibrosis across the liver section (Figure 5D,F and Figure 6I–L).

An effect of vitC status on hepatic histopathology was not recorded in the HF groups. LF intervention groups (HFL/LFL and HFL/LFH) were not statistically different; however, an effect of vitC was indicated as HFL/LFH animals decreased fibrosis, inflammation, and ballooning cells compared with both HF intervention groups (HFL/HFH and HFL/HFL; *p* < 0.05 or less), whereas this was not as clear in the HFL/LFL group, where animals appeared to exhibit increased scoring on several parameters (Figure 5B,C).

### 3.5. Transcriptome Analysis

**Figure 7:**
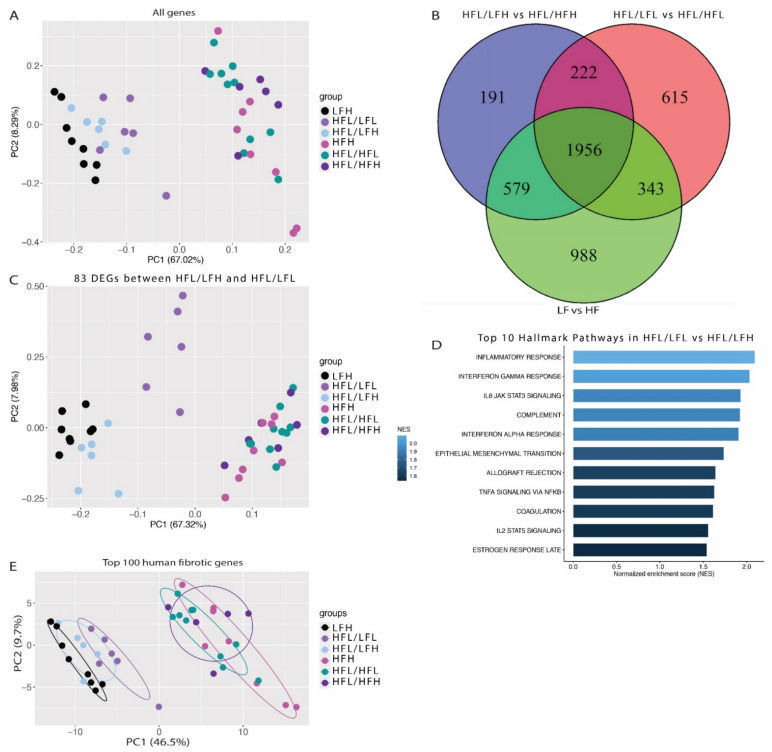
Transcriptomic differences between groups. (**A**) Principal component analysis (PCA) plot based on normalized and transformed values for all genes in all investigated samples. (**B**) Venn diagram of differentially expressed genes between HFH and LFH; HFL/HFH and HFL/LFH; and HFL and HFL/LFL, showing overlapping and unique genes for each dataset. (**C**) PCA plot using the 83 differentially expressed genes between HFL/LFL and HFL/LFH. (**D**) Top 10 regulated pathways in HFL/LFL vs. HFL/LFH. The color bar depicts normalized enrichment score. Only significantly upregulated pathways are included (*p* < 0.1) (**E**) PCA plot using the top 150 differentially expressed genes from the human NAFLD/NASH dataset [54]. Encircled areas represent the normal data distribution within a 68% confidence interval. HF: all high fat fed groups; HFH: high fat high vitC, HFL: high fat low vitC, LF: all low fat fed groups; LFL: low fat low vitC, LFH: low fat high vitC, VitC: vitamin C.

RNA-sequencing was performed on all (LFH (*n* = 8), HFH (*n* = 8), and HFL/HFL (*n* = 10)) or a randomly selected subset of animals (HFL/LFH, HFL/LFL, and HFL/HFH, *n* = 6). Principle component analysis (PCA) showed clear separation of all groups based on diet (Figure 7A), and showed a total of 1956 differentially expressed genes in common between groups receiving a control diet and their respective HF counterparts (Figure 7B).

Differential gene expression analysis was performed to assess if the slightly improved histopathology in the HFL/LFH animals compared with the HFL/LFL group was reflected by changes in the hepatic transcriptome. This resulted in the identification of 83 DEGs between HFL/LFH and HFL/LFL (see Supplementary Table S4 for a complete list of identified genes). PCA of these genes in the remaining groups revealed a grouping of animals from the HFL/LFL group, indicating a similar expression pattern in all HF groups and, importantly, in the LFH (control) and HFL/LFH group compared with HFL/LFL animals (Figure 7C). Gene set enrichment analysis highlighted an upregulation of inflammatory pathways in HFL/LFL compared with HFL/LFH (Figure 7D). Finally, the expression pattern of the top 150 fibrotic DEGs derived from a human dataset of NAFLD/NASH patients [54] resulted in a distinct separation of all HF and LF diet groups. As indicated in Figure 7E, samples from the three LF groups appear to cluster in three separate bands, with the LFH (control) group furthest away from the HF groups, closely followed by the HFL/LFH group, and finally the HFL/LFL group closest to the HF cluster. The complete RNA sequence data set can be found through GSE 192497.

## 4. Discussion

This study investigated the effects of vitC on the progression and potential resolution of NASH following dietary induction and subsequent intervention in the guinea pig disease model. NASH with fibrosis was evident in induced groups regardless of vitC supplementation, confirming the model and the high fat/high cholesterol diet as the main driver of the disease. Importantly, following a change to a low fat/low cholesterol diet, a positive effect of vitC status on the NASH transcriptome during regression was recorded, suggesting a beneficial role of this antioxidant in supporting NASH treatment strategies.

### 4.1. Low Fat Diet Drives Resolution of NASH

Hepatic histopathology—steatosis, ballooning, and NAFLD activity score—improved in both LF intervention groups (HFL/LFH and HFL/LFL) to levels comparable to LF animals and below week 16 (pre-intervention) status of HFL counterparts, supporting NASH regression rather than a delayed progression during intervention. This was not as clear for the distribution of fibrosis, likely owing to the chronic nature of fibrotic scarring compared with the more dynamic response, e.g., of inflammatory cells, but limiting conclusions of fibrosis resolution as opposed to delayed progression. Post-intervention, both HFL/LFH and HFL/LFL groups displayed a lower fibrosis fraction compared with the HFL/HFL group, and approaching pre-intervention levels. Fibrosis fraction in HFL/LFH animals was also decreased compared with HFL/HFH. The semi-quantitative scoring of liver histopathology did not unequivocally support these differences; however, the pathological assessment is based on the morphological distribution of fibrosis within the hepatic parenchyma—such as the presence or absence of fibrotic bridging—aimed for diagnostic hallmarks, and is a descriptive methodology as opposed to quantifiable measures. Hence, the recorded reductions in overall liver fibrosis may not yet have reached a sufficient degree to be reflected in significant alterations of general histopathology. An improvement in hepatic health is supported by circulating enzymes ALT and AST with levels comparable to LFH controls in both LF intervention groups, whereas the high fat fed groups show a significant increase. The recorded increase in AST and ALT in HFL/HFH animals compared with the HFL/HFL group is encompassed with a very large variation for unknown reasons, and may not be directly reflective of the hepatic status in these animals.

### 4.2. Vitamin C Deficiency Delays Recovery of the NASH Transcriptome

However, fibrosis quantification and RNA-sequencing results indicated a slightly superior effect of receiving the low fat/low cholesterol diet in combination with a high vitC level in diminishing NASH. The PCA of all genes demonstrated a separation of groups based on dietary fat and cholesterol and showed that the reversion to a normalized and healthy hepatic profile was dynamic and occurred after a relatively short intervention period. The second PCA plot showed similar regulation of the 83 identified DEGs in LFH and HFL/LFH groups, compared with the HFL/LFL group, indicating that the transcriptomic profile of LFH animals more closely resemble that of HFL/LFH rather than HFL/LFL. This is perhaps not surprising as the two diets are alike. However, the HF groups do not separate clearly and display no difference due to vitC levels between intervention groups (HFL/HFH vs. HFL/HFL). Furthermore, the span between the transcriptomic profile of fibrotic genes in HFL/LFH and HFL/LFL indicates that NASH fibrosis is increased in vitC deficient animals. Compared with the parallel high fat intervention groups (HFL/HFH and HFL/HFL), this indicates that these changes in the hepatic transcriptome are linked to disease resolution (driven by a low fat diet) rather than delayed disease progression, supporting a beneficial effect of an LFH diet compared with an LFL diet on the recovering NASH transcriptome.

Interestingly, C-reactive protein is among the 83 identified DEGs between HFL/LFH and HFL/LFL groups, and is upregulated in the HFL/LFL group by a log 2 fold change of 1.78. In humans, C-reactive protein is an inflammatory marker linked to obesity and atherosclerosis [55,56,57], and more recently also to NAFLD [58,59,60]. Guinea pig C-reactive protein shares sequence and subunit homology with humans, confirming conservation of the protein, but is not characterized as an acute phase inflammatory reactant in vivo in this species [61,62]. However, gene set enrichment analysis of hallmark pathways revealed an overrepresentation of pathways involved in inflammatory signaling, which could indicate that the hepatic inflammatory signaling profile is improved in response to vitC supplementation. The gene encoding collagen type IV alpha 2 chain (COL4A2) was among the 83 DEGs identified and represented in the top 150 human fibrotic genes in NAFLD [54]. The Collagen IV subunit is a major constituent of a fibrous extracellular matrix and basement membranes, and is increased in mild and severe hepatic fibrosis [63]. We have recently identified a list of genes highly associated with fibrosis in the guinea pig NASH model with severe hepatic fibrosis and with similar expression pattern in transcriptomic data from NAFLD patients [34]. This list included carbohydrate sulfotransferase 11 (CHST11), also significantly upregulated in HFL/LFL compared with HFL/LFH in the present study, supporting an increased fibrotic gene expression in the HFL/LFL group. Corroborating these observations, principle component analysis of the top 150 human fibrotic genes revealed a marginally closer relationship between fibrotic gene regulation in LFH and HFL/LFH compared with the HFL/LFL group, indicating that the fibrotic gene expression pattern in the HFL/LFH group was closer to complete reversal than the HFL/LFL group.

Previous studies in guinea pigs on high fat or cholesterol diets combined with vitC deficiency have reported changes in hepatic lipid-metabolism, e.g., increased hepatic TG and cholesterol and hepatocellular necrosis and fibrotic tissue expansion (in severely deficient animals) compared with non-deficient counterparts [64,65]. A recent study in senescence marker protein 30 (SMP30) knockout mice devoid endogenous vitC synthesis reported that vitC deficient (vitC serum concentration below 2.5 µg/mL) failed to progress from simple steatosis to more advanced disease compared with wild type counterpart mice on a high fat diet during 11 weeks of study [66]. The authors propose that a chronic state of vitC deficiency may mediate cholesterol build-up and subsequent inhibition of de novo lipogenesis, in turn delaying NAFLD progression. Although this supports a role of vitC in NAFLD, e.g., through a role as a cofactor for CYP7A1 and bile acid metabolism [67,68], no differences in hepatic TC levels were detected between HFH and HFL/HFL groups, hence no apparent effect of vitC status on hepatic cholesterol content in the present study.

### 4.3. Study Limitations

It may be speculated that the effect of high fat diet used in our study overpowered any effects of vitC deficiency. This is supported by RNA-sequencing that revealed grouping of all HF fed animals in the principle component analysis regardless of vitC status. It should be noted that the caloric intake in the HFL group was smaller during the 16 weeks pre-intervention period compared with counterparts. Although energy intake was equal between groups at post-intervention, the body weight curve of HFL/HFL animals appeared closer to LF groups (LFH, HFL/LFH, and HFL/LFL) compared with HFL/HFH and HFH animals. An initially reduced energy intake may have delayed induction of metabolic dysfunction and NASH progression in this group, leading to a reduction of the expected effects. This is likely also reflected in the OGTT responses, in which HFH—but not HFL groups—displayed a compromised glucose homeostasis compared with LF groups, supported by the overall effect of diet as a factor. Post-intervention, LF groups (HFL/LFH and HFL/LFL) show a return to a normalized metabolic competence. A high fat diet has previously been reported to suppress insulin tolerance in guinea pigs [69]; however, the ITT responses in the current study were not significantly different between groups, apart from a reduction in the HFL/HFL group at week 32. This may be due to the reduced weight gain in this group, but could also be caused by a difference in insulin production. Unfortunately, there are currently no commercially available detection methods for measuring insulin in guinea pigs, preventing further elaboration on this aspect. The increase of hepatic markers in HFL/HFH animals at week 32 is likely to be driven primarily by outliers and not directly linked to NASH. However, further exploration is required to determine if this effect is reproducible and reflective of a biologically relevant effect. Unfortunately, this could not be pursued in the current study owing to a lack of appropriate samples.

### 4.4. Final Remarks

While controlled clinical studies are yet to assess a role of vitC on the progression and regression of NAFLD/NASH, epidemiological data suggest a lower dietary vitC intake in NAFLD patients compared with healthy controls, linking vitC to disease propagation [23,24]. A beneficial effect of supportive antioxidant therapy in NASH is underlined by current guidelines that dictate lifestyle modifications as the primary treatment for NAFLD, while patients diagnosed with NASH may also be considered for vitamin E supplementation (800 IU/day) [12,13,14]. In line with this, the results presented in this study suggest that adequate vitC intake in addition to a diet change toward a low fat/low cholesterol regime has a beneficial effect in the treatment of NASH compared with dietary intervention alone. Thus, future clinical studies should explore if vitC supplementation in addition to lifestyle modifications may accelerate disease regression in recovering NASH patients with poor vitC status.

## 5. Conclusions

In conclusion, we show that vitC deficiency delayed hepatic improvements compared with animals with a sufficient vitC intake when switching from HF to LF diet, indicating a beneficial role of vitC in the regression of NASH. In contrast, vitC deficiency had a limited effect on NASH progression, though beneficial effects may be masked by the severity of lipotoxic lipids (cholesterol) driving the disease.

## Figures and Tables

**Table 1:** HbA1c plasma levels in all groups at week 32.

	LFH	HFH	HFL/HFL	HFL/HFH	HFL/LFH	HFL/LFL	Diet	VitC	Diet/vitC
**HbA1c**	3.90 ±0.13	4.25 ± 0.22 **	4.20 (3.80–4.23)	4.20 (4.18–4.40) ***	3.90 (3.90–3.93) ^†††^	3.90(3.80–4.00)	¤¤¤	ns	ns

Data are presented as medians with Q25–Q75 (in brackets) or means ± SD and analyzed by two-way ANOVA with a Tukey’s test for multiple comparisons, or a one-way ANOVA (all groups compared to LFH) (*n* = 8–10/group). Difference from LFH: ** *p* < 0.01, *** *p* < 0.001; difference from HFL/HFH: ^†††^ *p* < 0.001. For overall effect of factor (diet, vitC, and diet/vitC) ¤¤¤ *p* < 0.001. ns: not significant. HbA1c: hemoglobin A1C, HFH: high fat high vitC, HFL: high fat low vitC, LFH: low fat high vitC, LFL: low fat low vitC, vitC: vitamin C.

**Table 2:** Plasma levels for all groups following 32 weeks on diet.

Plasma Marker	LFH	HFH	HFL/HFL a	HFL/HFH b	HFL/LFH c	HFL/LFL d	Diet	VitC	Diet/VitC
VitC ^1^ µmol/L	48.17 (34.91–58.63)	33.92 (21.89–39.55)	1.76 (1.40–1.92) ***^b,c^	34.51(25.76–46.87) ^a,d^	31.82 (27.59–36) ^a,d^	1.74 (1.61–1.84) ***^b,c^	ns	¤¤¤	ns
FFA mmol/L	0.38 ± 0.22	0.54 ± 0.14	0.5 ± 0.14	0.53 ± 0.18	0.57 ± 0.17	0.63 ± 0.20	ns	ns	ns
TG ^1^ mmol/L	0.70 ± 0.19	0.54 ± 0.11	0.64 (0.53–0.69)	0.46 (0.37–0.59) ^*d^	0.46 (0.39–0.57)	0.62 (0.52–0.93)	ns	¤¤	ns
TC ^1^ mmol/L	0.53 (0.43–0.66)	7.42 (5.94–7.84) ^***^	6.88 (6.18–8.97) ^***c,d^	6.01 (4.92–7.73) ^***c,d^	0.43 (0.37–0.55) ^a,b^	0.63(0.58–0.67) ^a,b^	¤¤¤	¤¤	ns
ALT ^2^ U/L	32.95 (25.18–36.95)	66.90 (48.9–93.5)	37.80 (35.2–49.45) ^b,c,d^	67.76 (58.83–114.5) ^**a,c,d^	22.85 (21.825–26.73) ^a,b^	26.30 (18.43–34.30) ^a,b^	¤¤¤	¤¤	¤¤¤
AST ^1^ U/L	31.85 (20.40–36.23)	469.8 (394.70–556.20)^***^	205.75 (153.78–389.48) ^***b,c,d^	589.95 (302.08–1011.55) ^***a,c,d^	31.40 (24.30–52.13) ^a,b^	27.60 (25.30–43.03) ^a,b^	¤¤¤	¤	ns
ALP ^3^ U/L	54.50 ± 8.02	43.00 ± 10.28 ^**^	42.50 (39.00–48.00) ^**^	39.5 (36.75–41.75) ^***c^	51 (42.25–55.75) ^b^	45.00 (41.00–49.75)	¤¤¤	ns	ns

^1^ Analyses were performed on log transformed data. ^2^ Comparison to LFH was analyzed by a Kruskal–Wallis test with a Dunn’s multiple comparisons test and comparison between intervention groups was analyzed by two-way ANOVA on log transformed data. ^3^ Comparisons between intervention groups were analyzed by two-way ANOVA on log transformed data. Data are presented as means ± SD or medians with Q25–Q75 values in brackets. LFH was compared to all groups by one-way ANOVA with a Dunnett’s test for multiple comparisons. Post-intervention groups (HFL/HFL; HFL/HFH; HFL/LFH; and HFL/LFL) were compared by two-way ANOVA with a Tukey’s test for multiple comparisons (*n* = 8–10/group). Difference from LFH: * *p* < 0.05, ** *p* < 0.01, *** *p* < 0.001. Differences between intervention groups are illustrated by different superscript letters **a, b, c**, and **d**: *p* < 0.05 or less. For overall effect of factors (diet, vitC, and diet/vitC) (two-way ANOVA): ¤ *p* < 0.05, ¤¤ *p* < 0.01, ¤¤¤ *p* < 0.001. ns: not significant. ALP: alkaline phosphatase, ALT: alanine aminotransferase, AST: aspartate aminotransferase, FFA: free fatty acids, HFH: high fat high vitC, HFL: high fat low vitC, LFL: low fat low vitC, LFH: low fat high vitC, TC: total cholesterol, TG: triglycerides, VitC: vitamin C.

**Table 3:** Liver levels of measured markers following 32 weeks on diets.

	LFH	HFH	HFL/HFL a	HFL/HFH b	HFL/LFH c	HFL/LFL d	Diet	VitC	Diet/VitC
**VitC ^1^** **nmol/g**	1682.00 (1497.00–1913.00)	1181.00 (1073.00–1426.00) *	128.20 (103.60–147.60) ***^b,c^	1198.40 (1015.30–1316.80) ^**a,d^	1344.80 (1031.60–1691.20) ^a,d^	119.50 (95.40–139.10) ^***b,c^	ns	¤¤¤	ns
**TG ^2^** **µmol/g**	6.10 ± 4.05	37.76 ± 6.81 ^**^	42.29 (39.86–47.86) ^***c,d^	46.94 (43.86–56.33) ^***c,d^	6.58 (5.09–10.72) ^a,b^	8.00 (6.89–14.29) ^a,b^	¤¤¤	ns	ns
**TC ^1^** **µmol/g**	4.10 (3.77–4.46)	27.28 (24.70–32.53) ^***^	31.58 (29.92–33.19) ^***c,d^	31.79 (28.87–33.15) ^***c,d^	4.57 (4.17–5.14) ^a,b^	4.69 (4.46–5.09) ^**a,b^	¤¤¤	ns	ns

^1^ Log transformed data were analyzed by two-way ANOVA with a Tukey’s test for multiple comparisons. ^2^ Comparison to LFH was analyzed by a Kruskal–Wallis test with a Dunn’s test for multiple comparisons, and comparison between intervention groups was analyzed by two-way ANOVA on log transformed data. Data are presented as means ± SD or medians with Q25–Q75 values in brackets (*n* = 8–10/group). LFH was compared to all groups by one-way ANOVA with a Dunnett’s test for multiple comparisons. Post-intervention groups (HFL/HFL; HFL/HFH; HFL/LFH; and HFL/LFL) were compared by two-way ANOVA with a Tukey’s test for multiple comparisons. Difference from LFH: * *p* < 0.05, ** *p* < 0.01, *** *p* < 0.001. Differences between intervention groups are illustrated by different superscript letters a, b, c, and d: *p* < 0.05 or less. For overall effect of factors (two-way ANOVA): ¤¤¤ *p* < 0.001. ns: not significant. HFH: high fat high vitC, HFL: high fat low vitC, LFL: low fat low vitC, LFH: low fat high vitC, TC: total cholesterol, TG: triglycerides, VitC: vitamin C.

## Data Availability

The RNA sequencing data are publicly available via GSE192497. Additional data and material supporting the conclusions of this article can be requested from the authors.

## Supplementary Materials

The following are available online at https://www.mdpi.com/article/10.3390/antiox11010069/s1; Table S1: Detailed diet composition; Table S2: Plasma markers following 16 weeks on diets (pre-intervention); Table S3: Liver markers following 16 weeks on diets (pre-intervention); Table S4: Complete list of identified genes (DEGs).
