# Supplementary material for: Vitamin C Deficiency May Delay Diet-Induced NASH Regression in the Guinea Pig
Source: Antioxidants (Basel). 2021 Dec 28;11(1):69. doi: 10.3390/antiox11010069 (PMC8772888; doi:10.3390/antiox11010069)
Supplement: Supplementary file 1 [file antioxidants-11-00069-s001.zip › Supplementary table S1.pdf]

Supplementary data

Table S1: Detailed diet composition

Product		LF 2000 mg/kg vitC	LF 100 mg/kg vitC	LF 0 mg/kg vitC	HF 2000 mg/kg vitC	HF 100 mg/kg vitC	HF 0 mg/kg vitC
**Alfalfa**	%	33	33	33	22	22	22
**Wheat**	%	3.86	4.41	4.44	10	10	10
**Barley**	%	10	10	10	-	-	-
**Wheat Bran**	%	15.55	15.55	15.55	-	-	-
**Sucrose**	%	-	-	-	14.83	15.41	15.38
**Inulin**	%	3	3	3	-	-	-
**Cellulose (Lignocellulose)**	%	4	4	4	4.6	4.6	4.6
**Sunflower meal**	%	5	5	5	3	3	3
**Soybean meal**	%	10.2	10.2	10.2	6	6	6
**Soybeans (full fat)**	%	1	1	1	2.6	2.6	2.6
**Soybean concentrate**	%	-	-	-	12	12	12
**Corn gluten feed**	%	8	8	8	-	-	-
**Amino acids**	%	0.65	0.65	0.65	0.5	0.5	0.5
**Vitamin/trace element premix**	%	1	1	1	1	1	1
**Choline Cl**	%	0.3	0.3	0.3	0.3	0.3	0.3
**Vitamin C, stabilized**	%	0.58	0.029	-	0.58	0.029	-
**Sugar beet pulp**	%	1.3	1.3	1.3	1	1	1
**Soybean oil**	%	1.2	1.2	1.2	0.5	0.5	0.5
**Cholesterol**	%	-	-	-	0.35	0.35	0.35
**Coconut oil, hydrogenated**	%	-	-	-	18	18	18
**Crude protein (=N x 6.25)**	%	17.1	17.1	17.1	16.9	16.9	16.9
**Crude fat**	%	3.8	3.8	3.8	20	20	20
**Crude fiber**	%	19.8	19.9	19.9	11.4	11.4	11.4
**NDF[1]**	%	32.3	32.4	32.4	-	-	-
**Crude ash**	%	7.9	7.9	7.9	6.6	6.6	6.6
**Starch**	%	13.3	13.7	13.7	7.9	7.9	7.9
**Sugar**	%	4	4	4	17.3	17.9	17.9

**Carbohydrates (NfE)**	%	41.1	41.1	41.1			
**ME (Atwater)[2]**	MJ/kg	11.2	11.2	11.2	16.8	16.8	16.8
**Kcal% Protein**		26	26	26	17	17	17
**Kcal% Fat**		13	13	13	45	45	45
**Kcal% CHO**		61	61	61	38	38	38
**Fatty Acids, % in diet**							
**C8:0**	-	-	-	-	1.06	1.06	1.06
**C10:0**	-	-	-	-	0.86	0.86	0.86
**C12:0**	-	-	-	-	8.22	8.22	8.22
**C14:0**	0.01	0.01	0.01	0.01	3.53	3.53	3.53
**C16:0**	0.64	0.64	0.64	0.64	2.23	2.23	2.23
**C18:0**	0.11	0.11	0.11	0.11	2.32	2.32	2.32
**C20:0**	0.01	0.01	0.01	0.01	0.03	0.03	0.03
**C16:1**	0.02	0.02	0.02	0.02	0.01	0.01	0.01
**C18:1**	0.67	0.67	0.67	0.67	0.44	0.44	0.44
**C18:2**	1.89	1.89	1.89	1.89	0.96	0.96	0.96
**C18:3**	0.35	0.35	0.35	0.35	0.22	0.22	0.22

[1]NDF: Neutral Detergent Fiber; fiber fraction that is included in the CHO (NfE). [2]Atwater may not accurately reflect the ME of high fiber diets for guinea pigs as this is currently not known. Consequently the Atwater has been estimated using ME for rats.. Diets containing 50 mg vitC/kg feed were obtained by mixing 0 and 100 mg vitC/kg feed. All feed were manufactured by Ssniff Spezialdiäten, Soest, Germany, and vitamin C content confirmed by post production analysis. CHO: Hydrated carbohydrates, LF: Low fat diet; HF: high fat diet, ME: Metabolizable Energy, NfE: Nitrogen free extracts, vitC: vitamin C.
