# Supplementary material for: Vitamin C Deficiency May Delay Diet-Induced NASH Regression in the Guinea Pig
Source: Antioxidants (Basel). 2021 Dec 28;11(1):69. doi: 10.3390/antiox11010069 (PMC8772888; doi:10.3390/antiox11010069)
Supplement: Supplementary file 1 [file antioxidants-11-00069-s001.zip › Supplementary table S2.pdf]

## Supplementary data

**Table S2:** Plasma markers following 16 weeks on diets (pre-intervention).

| Plasma | LFH | HFH | HFL |
|---|---|---|---|
| VitC[1] µmol/L | 38.93(34.44-46.00) | 34.28 (26.26-41.91) | 1.98 (1.89-2.03)****,#### |
| FFA mmol/L | 0.55±0.07 | 0.61±0.09 | 0.62±0.26 |
| TG[1] mmol/L | 0.64(0.57-0.82) | 0.70(0.50-0.85) | 0.68(0.49-1.02) |
| TC[1] mmol/L | 0.52(0.49-0.85) | 7.12(4.98-11.23)**** | 5.55(4.28-6.94)**** |
| ALT[1] U/L | 29.70 (20.20-35.80) | 44.00(33.78-62.90) | 59.20(38.70-77.60)* |
| AST[1] U/L | 125.6(30.70-328.60) | 398.30(168.95-1002.65) | 368.30(320.60-532.80) |
| ALP U/L | 56.40±12.73 | 49.88±7.45 | 61.71±10.05 |

[1]Analysis was performed on log transformed data. Data are presented as medians with Q25-Q75 (in brackets) or means ± SD and analyzed by one-way ANOVA with a Tukey's test for multiple comparisons (n=7-8/group). Difference from LFH: *p<0.05, **p<0.01, ***p<0.001; Difference from HFH: ####p<0.0001. ALP: Alkaline Phosphatase, ALT: Alanine Aminotransferase, AST: Aspartate Aminotransferase, FFA: Free Fatty Acids, HFH: High Fat High vitC, HFL: High Fat Low vitC, LFH: Low Fat High vitC, TC: Total Cholesterol, TG: Triglycerides, vitC: Vitamin C.
