# Supplementary material for: Vitamin C Deficiency May Delay Diet-Induced NASH Regression in the Guinea Pig
Source: Antioxidants (Basel). 2021 Dec 28;11(1):69. doi: 10.3390/antiox11010069 (PMC8772888; doi:10.3390/antiox11010069)
Supplement: Supplementary file 1 [file antioxidants-11-00069-s001.zip › Supplementary table S3.pdf]

Supplementary data

Table S3: Liver markers following 16 weeks on diets (pre-intervention)

Liver	LFH	HFH	HFL
VitC[1] nmol/g	1299.0(1120.0-1572.0)	1108.0 (1023.0-1316.0)	153.2 (125.8-165.6)****,####
TG µmol/g	9.16±4.96	54.79±9.08****	61.41±9.41****
TC[1] µmol/g	4.31(3.91-5.06)	32.01(30.43-36.28)****	32.05(29.42-32.32)****

[1]Analysis was performed on log transformed data. Data are presented as medians with Q25-Q75 (in brackets) or means ± SD and analyzed by one-way ANOVA with a Tukey's test for multiple comparisons (n=7-8/group). Difference from LFH: ****p<0.0001; Difference from HFH: ####p<0.0001. HFH: High Fat High vitC, HFL: High Fat Low vitC, LFH: Low Fat High vitC, TC: Total Cholesterol, TG: Triglycerides, vitC: Vitamin C.
